# Non-Invasive Imaging of Prosthetic Heart Valves with a Focus on Endocarditis

**DOI:** 10.5334/jbr-btr.1056

**Published:** 2016-02-16

**Authors:** Ricardo P.J. Budde

**Affiliations:** 1 Erasmus Medical Centre Rotterdam, NL

Prosthetic heart valve (PHV) implantation can be a life-saving intervention. However, it does provide the patient with a lifelong chronic condition. Prosthetic heart valves can become dysfunctional due to various causes. The most dreaded complication is PHV endocarditis. Patients with a PHV are at higher risk to develop endocarditis because of the exposure of foreign material to the bloodstream. The reported incidence of PHV endocarditis varies considerably in the literature [1], probably because PHV endocarditis is such a difficult diagnosis to establish. The patient can present with a variety of symptoms that are often non-specific. Diagnosis of endocarditis has relied on the Duke criteria, which incorporates findings from echocardiography, physical examination, and blood cultures. The Duke criteria are known to be less accurate in PHV endocarditis [1]. Echocardiography is often hampered by PHV-induced artifacts, and blood cultures are more often negative.

In the last decade, CT imaging has emerged as a technique that can provide valuable information on the function of PHVs [2]. It provides information about leaflet motion as well as the structural integrity of the valve and surrounding cardiac anatomy. In case of PHV endocarditis, CT can demonstrate mycotic aneurysms that are especially frequent in the aortic position. The relationship between such mycotic aneurysms and the coronary arteries can also easily be assessed. CT is however less accurate in demonstrating abscesses surrounding the valve that have not yet established a connection with the bloodstream.

Positron emission tomography (PET) with labelled 18F-fluorodeoxyglucose(FDG) imaging has proven to be a valuable addition in the work up of PHV endocarditis in combination with CT imaging. It can demonstrate that areas of high metabolic turnover are present in infected tissue. Since the normal myocardium also takes up glucose, and thus FDG, a special preparatory diet is required to suppress normal myocardial metabolism to clearly distinguish FDG uptake as a result of infected tissue from normal myocardial uptake.

In the most recent guidelines of the European society of cardiology, the roles of CT and PET imaging have been clearly acknowledged by incorporating them into Duke criteria [1]. The total literature on PET imaging of PHV endocarditis is however still limited. Therefore, its’ exact role in the diagnosis of PHV endocarditis will likely become more clear in the next few years.

It is important that cardiovascular radiologists are aware of the possibilities of CT and PET imaging for diagnosing PHV endocarditis since they play an important role in establishing or rejecting the diagnosis which has a major clinical impact.

## Competing Interests

The author declares that they have no competing interests.
